# Antimicrobial effect of plantago major extract against *streptococcus mutans* in dental caries formation

**DOI:** 10.1590/0103-644020256538

**Published:** 2026-01-09

**Authors:** Heide Mendonça Moreira De Souza, Ivana Suffredini Barbosa, Karen Cristina Comin Maldonado, Cristina Lúcia Feijó Ortolani

**Affiliations:** 1 Universidade Paulista - UNIP, São Paulu-SP. Brazil

**Keywords:** Streptococcus mutans, phytotherapy, plantaginaceae

## Abstract

This study evaluated the efficacy of Plantago major extract against *Streptococcus mutans*, a bacterium associated with biofilm formation and the development of dental caries, in search of natural alternatives to conventional methods of caries control. An experimental design was conducted using bioautography and broth microdilution techniques to assess the antimicrobial activity of the extract, with chlorhexidine serving as the comparative substance. The results demonstrated that Plantago major consistently produced inhibition zones with a mean diameter of 9.95 ± 0.41 mm, although smaller than those observed for chlorhexidine (CHX 1%) and CHX 0.12%, which averaged 14.98 ± 0.22 mm. Statistical analysis confirmed the significance of the antimicrobial effect of the extract (p < 0.05), suggesting its potential as a natural therapeutic agent. In conclusion, Plantago major extract showed promise as an alternative strategy for controlling dental caries, presenting a lower risk of side effects. These findings encourage further investigation into phytotherapeutic approaches and support the advancement of clinical research aimed at improving oral health and addressing the growing challenge of antimicrobial resistance.

## Resumo

Este estudo avaliou a eficácia do extrato de Plantago major contra Streptococcus mutans, uma bactéria associada à formação de biofilme e ao desenvolvimento de cárie dentária, em busca de alternativas naturais aos métodos convencionais de controle da cárie. Um delineamento experimental foi conduzido utilizando técnicas de bioautografia e microdiluição em caldo para avaliar a atividade antimicrobiana do extrato, com clorexidina como substância comparativa. Os resultados demonstraram que o extrato de Plantago major produziu consistentemente zonas de inibição com diâmetro médio de 9,95 ± 0,41 mm, embora menores do que as observadas para clorexidina (CHX 1%) e CHX 0,12%, que apresentaram média de 14,98 ± 0,22 mm. A análise estatística confirmou a significância do efeito antimicrobiano do extrato (p < 0,05), sugerindo seu potencial como agente terapêutico natural. Em conclusão, o extrato de Plantago major mostrou-se promissor como uma estratégia alternativa para o controle da cárie dentária, apresentando menor risco de efeitos colaterais. Essas descobertas incentivam novas investigações sobre abordagens fitoterápicas e apoiam o avanço da pesquisa clínica voltada para a melhoria da saúde bucal e o enfrentamento do crescente desafio da resistência antimicrobiana.

**Figure:**
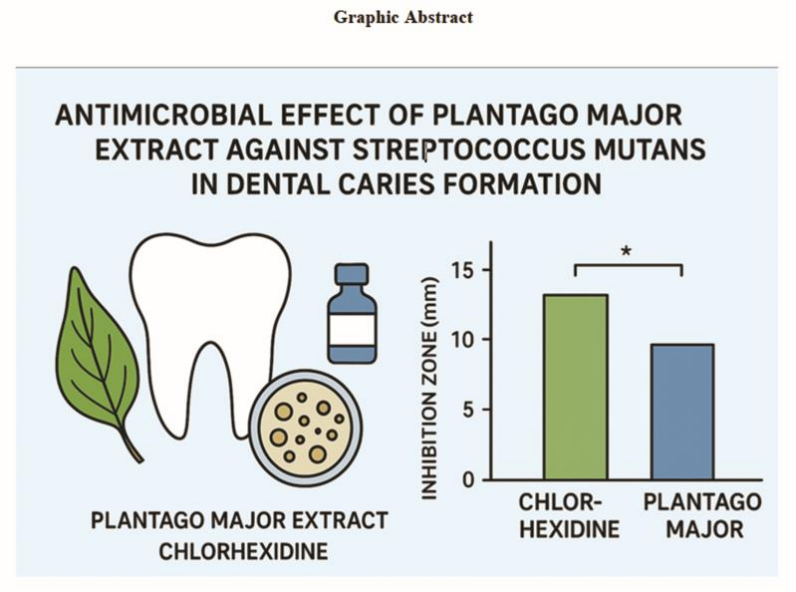

## Introduction

The oral microbiota consists of approximately 700 species of prokaryotes, distributed across 185 genera and 12 phyla, of which 54% have an official name, 14% are anonymous (cultivable), and 32% are known only as uncultivable phylotypes^1^. The development of dental caries is associated with a complex microbial community that adheres to the tooth surface, forming dental biofilm^2^. This biofilm is a multilayered system composed of bacteria, bacterial products, and saliva in a hydrated state, with distinct mechanical and rheological properties^3^. Species of the *Streptococcus* genus, especially *Streptococcus mutans* (S. mutans), play a crucial role in biofilm formation and the production of extracellular polysaccharides from sucrose^4^. Studies indicate that *S. mutans* also promotes the adhesion of other harmful bacteria^5^ ^,^ ^6^.

The control of dental caries represents a significant challenge for global public health, impacting quality of life and productivity. According to the World Health Organization^7^, dental caries affects approximately 60 to 90% of school-aged children and nearly 100% of adults worldwide^8^ ^,^ ^9^ ^,^ ^10^. This condition impacts chewing, growth, and development, in addition to affecting emotional well-being. Moreover, it can lead to permanent tooth loss^11^ ^,^ ^12^.

The initial phase of dental caries is characterized by white spot lesions. In recent years, an increase in prevalence has been noted, with rates ranging from 10% to 49%. These lesions are frequently found in patients undergoing orthodontic treatment and are caused by plaque accumulation in the cervical region of the teeth^13^. The use of fluoride in various forms remains the cornerstone of dental caries prevention. Fluoride primarily acts by inhibiting enamel demineralization and enhancing remineralization; it promotes the formation of acid-resistant fluorapatite that reinforces the enamel’s resistance to acid attack^14^. However, current methods, such as the addition of fluoride to the water supply, are considered insufficient. Therefore, additional approaches are necessary to improve treatment efficacy^15^

Dental biofilm can be mechanically controlled through practices such as brushing and flossing. Additionally, chemical approaches, mainly using substances like chlorhexidine, have been extensively studied due to their effective antimicrobial action^16^. For example, chlorhexidine (CHX) is a cationic bisbiguanide antiseptic that binds to negatively charged bacterial cell membranes, causing membrane disruption and leakage of cytoplasmic components^4^. At higher concentrations or with prolonged exposure, CHX causes cytoplasmic coagulation and precipitation of intracellular compounds, resulting in irreversible bacterial cell death^5^. Its positive charge also enables it to adsorb to oral surfaces, interfering with bacterial adhesion and inhibiting biofilm formation^6^. Although chlorhexidine is considered the gold standard for bacterial control, prolonged use can produce adverse effects, including taste alterations (dysgeusia), tooth staining, and, in rare cases, allergic reactions^17^ ^,^ ^18^ ^,^ ^19^. Furthermore, bacterial resistance to chlorhexidine is a growing concern, highlighting the need to explore safe and effective alternatives^20^.

In this context, herbal medicines have emerged as promising alternatives for controlling dental biofilm and preventing dental caries. They offer advantages such as lower toxicity, reduced cost, and easy accessibility^21^ ^,^ ^22^ ^,^ ^23^. However, the safety and efficacy of phytotherapeutic agents depend on factors such as dosage, preparation method, and individual patient response; some herbal constituents may be toxic at high concentrations or if improperly formulated^8^. Among the medicinal plants studied, *Plantago major* L. (Plantaginaceae), commonly known as Broadleaf Plantain, has drawn attention for its antibacterial, anti-inflammatory, and antioxidant properties^24^. *Plantago major* (P. major) is a plant known for its abundance of bioactive compounds, including flavonoids, terpenoids, iridoid glycosides, and fatty acids^25^. These compounds confer anti-inflammatory, wound-healing, and antioxidant properties to the plant, along with significant antibacterial activity. Studies have indicated that aucubigenin, derived from iridoid glycosides and present in Broadleaf Plantain, exhibits antibacterial activity, especially against gram-positive microorganisms such as *Streptococcus mutans* ^26^. In addition to these general effects, *P. major* contains flavonoids and phenolic compounds that can directly interfere with bacterial physiology. Flavonoids in *P. major* have been reported to intercalate into bacterial DNA and RNA, inhibiting nucleic acid synthesis and altering membrane fluidity. Tannins from the extract can bind to bacterial adhesins and enzymes, impairing *S. mutans* adhesion and metabolic activity. These multi-target actions likely contribute to the observed inhibition of *S. mutans* growth and cariogenic biofilm formation by the extract. ^27^

The present study aimed to evaluate the efficacy of *P. major* as an antibacterial agent against *Streptococcus mutans*, one of the primary pathogens involved in dental caries. The hypothesis of this study is that the extract of *Plantago major* L. possesses antimicrobial activity against *Streptococcus mutans*, potentially serving as an effective alternative in the prevention of dental caries, with potential applications in contemporary dentistry.

## Materials and Methods

An *in vitro study* was conducted to evaluate the antimicrobial activity of aqueous and ethanolic extracts of Plantago major L. against *Streptococcus mutans*. The minimum inhibitory concentration (MIC) and the minimum bactericidal concentration (MBC) were determined using the broth microdilution test, according to the guidelines of the Clinical and Laboratory Standards Institute (CLSI, 2008)^28^, with adaptations.

Antimicrobial activity was also investigated through the bioautography test associated with thin-layer chromatography, allowing the identification of inhibition zones and the evaluation of the extracts' efficacy. Both methods provided complementary data for characterizing the antimicrobial action of the tested extracts.

### Study Site

The present study was conducted at the Biodiversity Research Center (NPBio) of Universidade Paulista (UNIP), São Paulo, Brazil.

### Plant Material and Controls

The Plantago major L. (Plantaginaceae) extract, obtained from aerial parts, was purchased from Heide Extratos Vegetais, Pinhais, Paraná, Brazil (batch 2168, manufactured on 04/27/2023, valid until 05/2025, quality control nº 84929-431). In addition, 1% and 0.12% chlorhexidine gluconate solutions provided by Fórmula & Ação, São Paulo-SP, Brazil (manufactured on 10/15/2023, valid until 10/2025) were used as positive controls.1.2 Preparation of Bacterial Inoculum

The *Streptococcus mutans* strain (ATCC 25175) was previously thawed and cultured on Brain Heart Infusion (BHI) agar (Oxoid®). A bacterial suspension was prepared in 0.9% saline solution, and the concentration was adjusted to 1.5 × 10⁸ CFU/mL for the pilot test and experiment 01^29^, 1.5 × 10⁷ CFU/mL for experiment 02, and 1.5 × 10⁶ CFU/mL for experiment 03. The concentrations were determined by viable colony counts through serial dilutions or by turbidity adjusted to the MacFarland scale.

Subcultures were generated from two master plates, each containing 100 μL of thawed bacterial culture. These subcultures were transferred to Petri dishes containing Oxoid® CM1136 BHI agar and incubated at 36°C for 48 hours^30^. A bacterial suspension was prepared from these subcultures by performing serial dilutions in seven tubes with 9 mL of saline solution each. After incubation, colonies were counted, and the average count was used to adjust the bacterial suspension concentration according to the MacFarland scale (0.5 MacFarland).

### Evaluation of Plantago major Extract

The antimicrobial activity was evaluated by the agar disk diffusion method. *S. mutans* colonies were suspended in sterile saline solution and inoculated onto Petri dishes containing Oxoid® CM1136 BHI agar using disks impregnated with the extract and controls. Inhibition zones were measured after 48 hours of incubation at 36°C. When inhibitory zones were identified, their dimensions were measured precisely using a caliper and expressed in millimeters, as documented in previous studies^31^. Two measurements were taken for each inhibition zone-one vertically and one horizontally-to ensure precision.

### Determination of minimum inhibitory concentration and minimum bactericidal concentration

Bacterial suspensions were incubated in BHI broth with different concentrations of the tested agents for 48 hours at 36°C. Turbidity was evaluated, and subcultures were performed.

A bacterial suspension in BHI broth was prepared from young *S. mutans* colonies at a concentration of 1.5 × 10⁷ CFU/mL, as described in section 1.3. In a 96-well U-bottom microplate, 190 µL of bacterial suspension was added to each well. 40 µL of plant extract or 1% chlorhexidine at different concentrations were added in triplicate^32^. The *P. major* extract was tested at concentrations ranging from 40 mg/mL to 0.3125 mg/mL, based on preliminary studies indicating this range as potentially effective against *S. mutans*. Chlorhexidine was tested at concentrations ranging from 1% to 0.12%, according to standard concentrations used in the literature.

Six wells were reserved for the negative control (culture medium only) and six wells for the positive control (culture medium with *S. mutans* inoculum)^33^. The microplates were incubated at 36°C for 48 hours. Turbidity was evaluated, and subcultures were performed from each well using 2 µL of supernatant, which were inoculated onto sterile BHI agar plates^30^.

### Bioautography

### Diffusion in Bioautography (DiB)

The ability of the *Plantago major* extract to inhibit bacterial growth was evaluated by bioautography, combining thin-layer chromatography with the disk diffusion test. The experiment was performed in triplicate for the extract samples and positive control (1% chlorhexidine).

Silica gel GF254 aluminum-backed plates (5 x 5 cm) were used. 10 µL of the plant extract or positive controls was applied per sample. Chromatograms were placed in 90 mm Petri dishes containing 5 mL of BHI agar previously inoculated with *S. mutans* (1.5 x 10⁸ CFU/mL for experiment 01 and 1.5 x 10⁷ CFU/mL for experiment 02). After gelification, 10 mL of BHI agar inoculated with the same bacterial concentrations was added.

Plates were incubated at 36°C for 48 hours. After this period, inhibition zones were identified using MTT viability dye (tetrazolium salt, 0.5 mg/mL). The inhibition zones were measured with a caliper in both horizontal and vertical directions.

### Statistical Analysis

Statistical analyses were performed using GraphPad Prism version 10.1.2.324. Data are presented as mean ± standard deviation (SD) and 95% confidence intervals (CI). Normality was assessed by Shapiro-Wilk and Kolmogorov-Smirnov tests (Shapiro-Wilk W = 0.9332, 0.9516 and 0.8788 for *P. major*, CHX 0.12% and CHX 1%, respectively; corresponding p = 0.415, 0.6602 and 0.2638; Kolmogorov-Smirnov KS = 0.1478, 0.1458 and 0.2361, p > 0.10 for all groups), indicating that the samples did not deviate from normality at α = 0.05. Homogeneity of variances was evaluated with Brown-Forsythe and Bartlett tests and showed no significant differences between groups. Outlier detection using the ROUT method (Q = 5%) identified no outliers. For comparisons among the three treatment groups (*P. major*, CHX 0.12% and CHX 1%), one-way ANOVA was used followed by Tukey’s multiple comparisons. Sample sizes for the DiB experiments were n = 12 (*P. major*), n = 12 (CHX 0.12%), and n = 6 (CHX 1%); these arise from two independent experiments with three disks per treatment and two orthogonal measurements per disk (totaling six measurements per treatment per experiment).

## Results

Determination of Minimum Inhibitory Concentration and Minimum Bactericidal Concentration**.** The determination of the minimum inhibitory concentration (MIC) was performed using the broth microdilution method, as described in the methodology. The MIC is the lowest concentration of the antimicrobial agent capable of interrupting the growth of the microorganism, reflecting its bacteriostatic capacity^34^. For the *S. mutans* strain, with an initial concentration of 1.5 × 10^7^ CFU/mL, the MIC of the extract was determined to be 25 mg/mL. This result indicates that, even at the highest dilutions tested, the extract maintained its inhibitory effect against the bacterium.

The 96-well plate microdilution tests confirmed the efficacy of the *Plantago major* extract in inhibiting the growth of *S. mutans*, with the MIC set at 25 mg/mL. Despite the 20-fold dilution in the microdilution technique, the extract demonstrated sustained antimicrobial activity, reinforcing the importance of the interaction between the chemical components of the extract for its efficacy. Although the MIC was established, the minimum bactericidal concentration (MBC) could not be determined with the tested concentrations, as even at the highest tested concentration of 40 mg/mL, no microorganism death was observed**.** In other words, under the conditions tested, *Plantago major* extract inhibited *S. mutans* growth (bacteriostatic effect) but did not kill the bacteria (no bactericidal effect). A bacteriostatic agent prevents bacterial proliferation (inhibiting growth) without causing irreversible cell death, whereas a bactericidal agent causes bacterial cells to die. After subculture, microbial growth was observed in all tested dilutions, confirming that the extract was bacteriostatic rather than bactericidal under the study conditions.

## Analysis and Results of Antibacterial Activity by Bioautography

**Figure 1:**
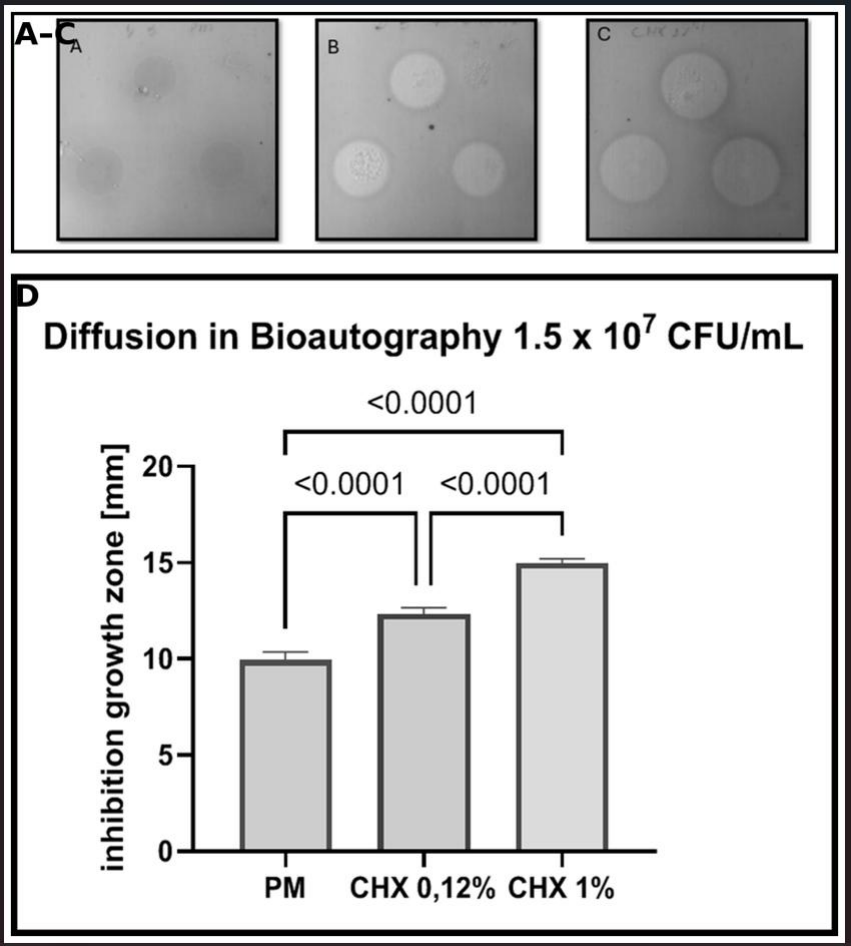
Antimicrobial activity of *Plantago major* extract and chlorhexidine measured by bioautography and diffusion assay.

The bioautography diffusion technique was used to measure the inhibition zones formed by P. major and chlorhexidine (CHX) treatments against S. mutans. The results are presented in Figures 1A, 1B, and 1C and include detailed statistical analysis by one-way ANOVA and Tukey’s multiple comparisons test. The bar graph presents the mean inhibition zones for treatments with P. major, 0.12% chlorhexidine (CHX 0.12%), and 1% chlorhexidine (CHX 1%), with the standard error of the mean (SEM) represented by error bars. Significant differences between treatments are indicated by p-values above the bars (p < 0.0001). The descriptive values for the experimental groups were as follows: CHX 1%, mean = 14.98 ± 0.22 mm; CHX 0.12%, mean = 12.35 ± 0.31 mm; and *P. major*, mean = 9.95 ± 0.41 mm.

One-way ANOVA revealed a significant difference among the groups (F^2^ ^,^ ^27^ = 449.8; p < 0.0001). Tukey’s post-hoc test confirmed statistically significant differences between all pairwise comparisons (p < 0.0001). The descriptive and comparative results are summarized in Table 1.

**Table 1:** Mean inhibition zones (mm) with descriptive statistics and Tukey's multiple comparisons for Plantago major (PM), 0.12% chlorhexidine (CHX 0.12%), and 1% chlorhexidine (CHX 1%).

Group	n	Mean (mm)	DP (mm)	Standard Error (mm)	CI 95% Bottom	CI 95% Top
P. Major	12	9.95	0.41	0.118	6.689	10.211
CHX 0.12%	12	12.35	0.31	0.089	12.153	12.547
CHX 1%	6	14.98	0.22	0.09	14.749	15.211

The Tukey-adjusted pairwise comparisons between the treatment groups (Plantago major, CHX 0.12%, and CHX 1%), including mean differences, 95% confidence intervals, and adjusted p-values, are shown in Table 2.

**Table 2:** Statistical comparison of inhibition zones among treatment groups.

Group	Mean difference (mm)	CI 95%	p Value
P. Major vs CHX 0.12%	-2.4	-2.744 0 to -2.053	<0.0001
P. Major vs CHX 1%	-5.03	-5.452 to -4.605	<0.0001
CHX 0.12% vs CHX 1%	-2.63	-3.053 to -2.207	<0.0001

### Statistical results for dib analysis

The statistical analysis of the study was expressed by means, 95% confidence intervals, and p-values adjusted by Tukey's test for the samples of *P. major*, chlorhexidine (CHX 0.12%), and chlorhexidine (CHX 1%). The bioautography diffusion analysis at a concentration of 1.5 x 10⁷ CFU/mL. Statistical tests were conducted to evaluate the normal distribution of the data. The Shapiro-Wilk test produced W values of 0.9332, 0.9516, and 0.8788 for *P. major*, CHX 0.12%, and CHX 1%, respectively, with associated p-values of 0.415, 0.6602, and 0.2638, indicating that the data passed the normality test at the 0.05 significance level. Similarly, the Kolmogorov-Smirnov test showed KS distances of 0.1478, 0.1458, and 0.2361, with p-values above 0.1000 for all samples, confirming the normality of the data. Outlier analysis by the ROUT method (Q = 5%) did not identify any outlier values among the *P. major*, CHX 0.12%, and CHX 1% samples.

The statistical results of the bioautography diffusion analysis (DiB) revealed significant insights into the antibacterial activity of *Plantago major* extract compared to 0.12% and 1% chlorhexidine (CHX). Means, 95% confidence intervals, and p-values adjusted by Tukey's test were used for analysis. Although studies in the literature have investigated a variety of natural extracts for antimicrobial activity, the superiority of chlorhexidine is often observed, reinforcing the continued need for natural antimicrobial agents to mitigate side effects associated with conventional antimicrobials ^36^ ^,^ ^37^. The results of the present study highlight the importance of Plantago major as a potential source of antibacterial compounds, with its inhibition halos averaging 9.95 mm.

## Discussion

The present study evaluated the antimicrobial effect of *Plantago major* extract against *Streptococcus mutans* and its capacity to reduce dental biofilm formation. Our results indicate that *P. major* produced significant inhibition of *S. mutans* growth and attenuated biofilm formation in vitro, findings that corroborate previous reports of antimicrobial activity for *P. major* and related phytotherapeutics^20^ ^,^ ^34^.

Mechanistically, the observed activity of *P. major* can be attributed to its complex phytochemical composition, which includes iridoid glycosides, flavonoids, and phenolic compounds. These constituents likely act through multiple, complementary routes: disruption of bacterial membrane integrity and membrane fluidity, inhibition of nucleic acid synthesis, and binding to bacterial adhesins and enzymes involved in adherence and extracellular polysaccharide production. Such multi-target effects plausibly impair *S. mutans* physiology and the organism’s ability to produce the extracellular matrix required for robust biofilm maturation ^20^ ^,^ ^23^ ^,^ ^25^. These mechanistic considerations are consistent with other in vitro reports demonstrating *P. major*’s antimicrobial and antibiofilm properties ^25^ ^,^ ^34^.

In the present study, the extract produced a measurable MIC (25 mg/mL), but an MBC was not demonstrable at the highest concentration tested (40 mg/mL). This result indicates a bacteriostatic action under the tested conditions: the extract inhibits bacterial proliferation without causing irreversible cell death at the concentrations assessed. Clinically, bacteriostatic agents can reduce microbial load and virulence, yet their effectiveness depends on adjunctive measures (host immune response, mechanical plaque removal, and topical fluoride), because residual viable cells may regrow if favorable conditions persist ^7^ ^,^ ^26^. Therefore, a bacteriostatic phytotherapeutic such as *P. major* would more appropriately be considered as a complementary strategy rather than a standalone replacement for established preventive measures.

Quantitative comparison in our bioautography assays showed larger inhibition zones for chlorhexidine than for *P. major*, reflecting the recognized in vitro potency of chlorhexidine ^16^. Nevertheless, chlorhexidine use is associated with adverse effects and tolerability concerns when used long-term (e.g., taste alteration, staining), and there are increasing discussions about resistance or reduced susceptibility following prolonged exposure ^17^ ^,^ ^18^. These issues motivate the search for adjunctive agents that may offer acceptable antimicrobial activity with different safety or tolerability profiles. In this context, *P. major* may provide antimicrobial benefit with a lower side-effect burden, but direct clinical comparisons require comprehensive evaluation of efficacy, safety, and long-term outcomes.

It is essential to emphasize that dental caries is a multifactorial disease: microbial colonization is only one determinant. Host factors (salivary flow and composition), dietary habits (frequency and quantity of fermentable carbohydrate intake), fluoride exposure, and the effectiveness of mechanical plaque control are all fundamental elements in caries risk and progression ^6^ ^,^ ^17^. Accordingly, any prospective role for *P. major* in caries control must be considered within an integrated preventive approach that addresses diet, fluoride use, and oral hygiene.

This study has several limitations that constrain direct clinical extrapolation. First, the work was performed in vitro using a single reference strain of *S. mutans* and therefore does not capture the ecological complexity of multispecies oral biofilms or host-microbe interactions ^3^ ^,^ ^23^. Second, the number of experimental replicates per group was limited, and the extract batch was not fully chemically standardized, factors that may affect reproducibility across laboratories ^25^ ^,^ ^34^. Third, safety assessments such as cytotoxicity or mucosal tolerance tests were not performed, so topical biocompatibility remains unknown. Fourth, while diffusion and dilution assays are established screening tools, translating MIC/MBC values into clinically effective formulations and concentrations requires pharmacokinetic, formulation, and in vivo evaluation^26^. Finally, although statistical analyses were applied to the present dataset, larger sample sizes and more robust experimental designs (e.g., blinded outcome assessment, pre-registered protocols) would strengthen future investigations.

To advance the evidence base, future research should: ^1^ use chemically standardized *P. major* preparations with complete phytochemical profiling; ^2^ evaluate antimicrobial and antibiofilm effects in multispecies biofilm models and in situ or in vivo systems that better mimic clinical conditions; ^3^ perform cytotoxicity and mucosal tolerance testing to assess safety for topical use; ^4^ explore potential synergistic interactions with fluoride or mechanical plaque control; and ^5^ design randomized clinical trials to determine clinical effectiveness in caries prevention or as an adjunct in periodontal or mucosal therapies. Methodological improvements such as larger sample sizes, blinded assessments, and robust statistical planning will increase reliability and reproducibility.

In summary, *Plantago major* extract demonstrated bacteriostatic antimicrobial activity against *S. mutans* and reduced biofilm formation in vitro. Given its multi-constituent mode of action and the global need for alternative antimicrobial strategies, *P. major* warrants further preclinical standardization and clinical evaluation as a complementary agent in oral care regimens. Nonetheless, current evidence supports a cautious interpretation: *P. major* is a promising adjunct but not yet a substitute for established preventive measures such as fluoride application, mechanical plaque control, and, when clinically indicated, short courses of conventional antimicrobials.

## Conclusion

The present study demonstrated the significant antimicrobial activity of *Plantago major* extract, evidenced by its efficacy in diffusion and microdilution tests against *Streptococcus mutans*, a critical dental pathogen. Although the inhibition zones were smaller than those produced by chlorhexidine, *P. major* extract proved to be a promising natural alternative, with the potential to combat biofilm formation and the progression of dental caries.
